# Surgery

**Published:** 1910-06

**Authors:** James Swain


### SURGERY.

**Injury to the Semilunar Cartilages of the Knee-joint** is a troublesome affection, which too often results in disappointment to the patient and practitioner. The subject is fully discussed in an excellent paper by Mr. Robert Jones,[3] based upon a personal experience of over five hundred operations. The wedge-shape of the cartilages practically determines the fact that only internal displacements can produce locking of the joint, and it is this internal displacement which gives rise to the acuteness of the attack.

[2] *Ibid.*, 1910, i, 460. [3] *Ann. Surg.*, 1909, l, 969.

The most common symptom is a sudden inability fully to extend the leg, and the usual cause of the mischief is a strain thrown on the internal lateral ligament while the knee is flexed and the femur rotated inwards. When the patient reaches home the knee is distended and manipulation difficult, and for several days there will be pain on pressure over the articular edge of the tibia opposite the ligament. If the patient is allowed to get about after two or three weeks' rest and massage, he is almost certain to get a relapse of the symptoms sooner or later, but the successive attacks to which he is liable are frequently not so severe as the primary one.

The localised pain produced by pressure over the injured spot is best elicited while the knee is flexed, when pressure between the bones can be more effectively made.

Diagnosis is by no means always easy, and in many cases only an exploration can give us accurate information. Hypertrophy of the synovial fringes, loose bodies, lipomata, osteomata and rupture of the crucial ligaments may be confused with a damaged semilunar cartilage. Injury of the internal semilunar cartilage is diagnosed by the acute onset, the persistence of pain on pressure over the injured area and the locking of the joint. The patient usually refers the pain to the front of the joint until pressure decides the real seat of the trouble. The history of the mode of production is helpful in arriving at a diagnosis. Injury to the external cartilage produces similar symptoms on the outer side of the knee, but is much more rare.

The symptoms of the nipping of a synovial fringe are less acute than those of displaced cartilage ; the pain is strictly local, and is not participated in by the internal lateral ligament. Creaking in the joint is a frequent accompaniment, and a swelling often occurs on each side of the ligamentum patellæ, due to the chronic thickening of the infra-patellar pad. Effusion always follows the nipping, but this is not an invariable rule with displacement of a semilunar cartilage

Loose bodies can usually be isolated by the patient, and the locking of the knee which they cause is only transitory. Effusions are common.

Lipomata sometimes lock the joint, causing swelling about the lower part of the patella and painless effusions. Pressure on the knee produces no pain, and the symptoms are rarely acute.

Osteomata may lock the joint when a muscle or tendon becomes entangled. They can be found by manipulation and by skiagraphy.

Rupture of the crucial ligaments allows the tibia to be moved in a to-and-fro direction, and when the knee is flexed the lateral movements are free. If the lateral ligaments are torn, lateral movements in the extended position are also free.

Mr. Jones maintains that a great distinction should be made between a primary and recurrent luxation of a semilunar cartilage, and that operation should only be performed on recurrent cases. The first displacement will not be accompanied by any degenerative changes, and the knee often recovers completely if appropriately treated. (*a*) Reduction must be absolute (*b*) All movements of the cartilage must be checked until union of the torn structures is complete. (*c*) No lateral strain must be allowed until the torn or stretched internal lateral ligament has recovered its tone and strength.

We can only be assured of reduction when the knee can be voluntarily held in complete extension. The best routine manipulation is acute flexion, lateral deviation and rotation inwards and full extension. Acute flexion is always painful, but it is the only position where internal rotation is most free; lateral deviation separates the bones which hold the cartilage, and full extension allows of readjustment and places the limb in such a position as to permit of accurate union of the internal lateral ligament. Retention of the cartilage in a fixed position can only be secured when the limb is fully extended, for the cartilages participate in all rotatory and lateral movements of the joint. This rest of the limb in a fully extended position should be maintained in bed as long as effusion lasts. When all fluid is absorbed we may proceed to the next stage. It may be days or weeks, and massage may help, but under no conditions must flexion be allowed. The third indication is to prevent for a prolonged period lateral deviation of the joint. Mr. Jones differs from Sir William Bennett[1] and Mr. Whitelocke[2] as regards the prevention of lateral strain, and considers that a short splint, devised merely to prevent flexion and lateral deviation for the first few weeks, offers the only logical hope of recovery to the injured structures. Afterwards massage, and exercises can be assiduously employed. One of the simplest methods of preventing strain upon the internal lateral ligaments is to walk upon an inverted foot with inturned toes, and in cases where the cartilage becomes displaced frequently Mr. Jones has the boots altered so that both heels and soles are raised on the inner side. When the patient stands in such a boot the foot is inverted, and the lateral strain which is needed to displace the cartilage is avoided.

In cases where operation is not advised or refused a splint is indicated, so devised as to prevent lateral strain upon the knee and to allow free movement. In all recurrent cases which do not need operation, and in all cases of first displacement sufficiently recovered for flexion to be allowed, one orders a splint, alters the boot for the purpose of inverting the foot, and disallows walking except with parallel feet and running except with

[1] *Injuries and Diseases of the Knee-Joint*, 1909.

[2] *Sprains and Allied Injuries to Joints*, 1909.

inturned toes. In addition to this, the quadriceps muscle must be well massaged.

Mr. Jones refuses to operate in cases seen early, the subject of a first derangement. He discourages operation in those recurrent cases where the symptoms are transient and not followed by irritation of the joint, but strongly urges it in those cases where a recurrent displacement is at times followed by acute symptoms. He advises it in all recurrent cases where a strenuous athletic life is a means of livelihood or a physical necessity, and regards operation as absolutely imperative in the case of men who work or stand in dangerous places and where yielding of the knee may lead to serious consequences.

JAMES SWAIN.

